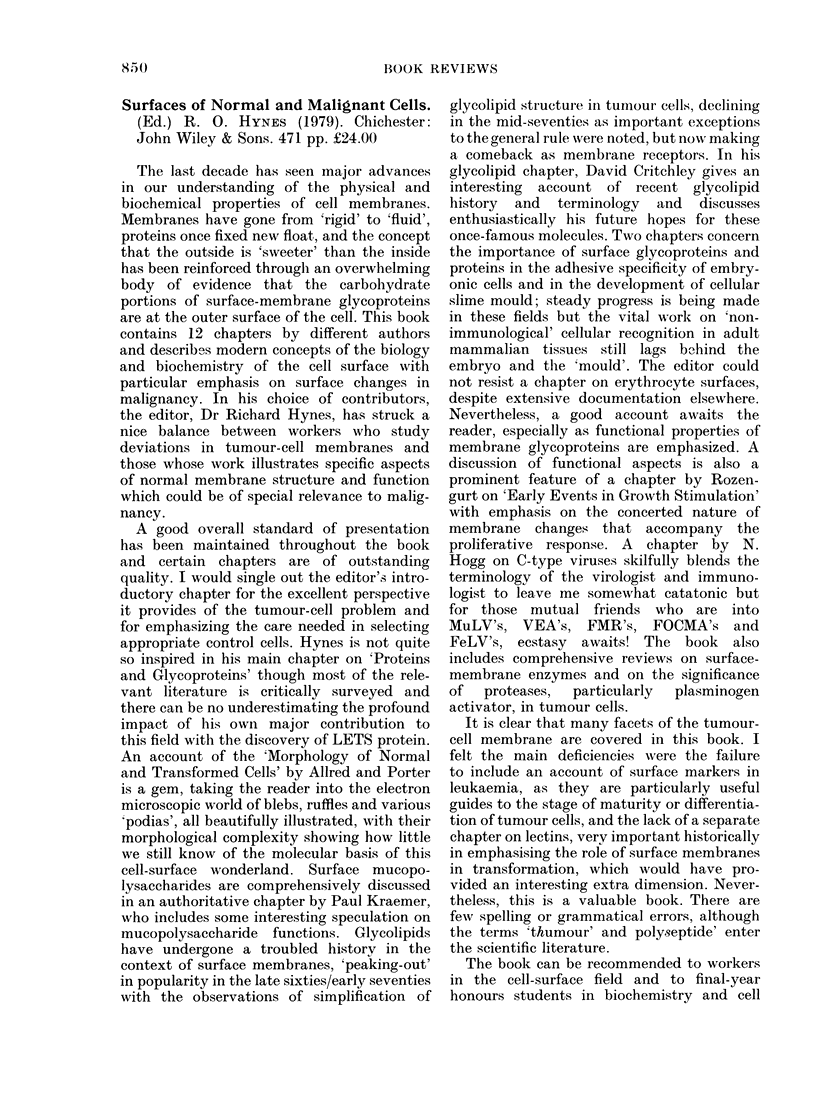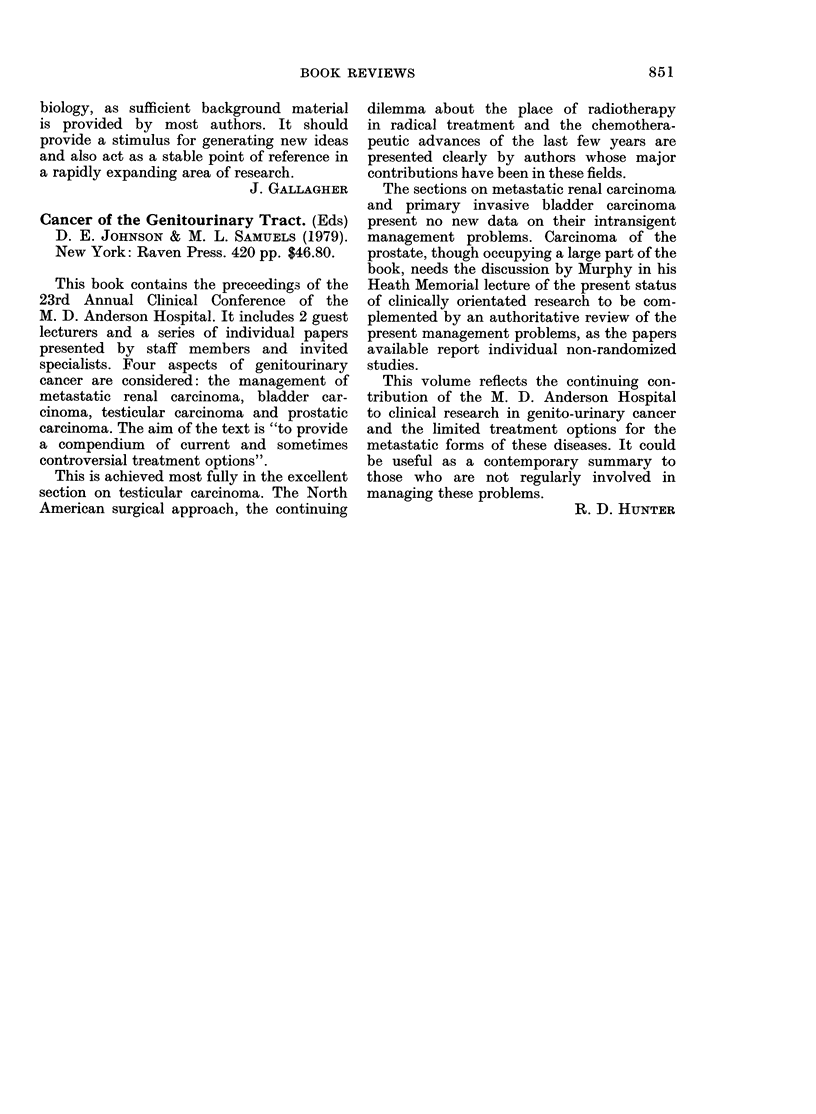# Surfaces of Normal and Malignant Cells

**Published:** 1980-05

**Authors:** J. Gallagher


**Surfaces of Normal and Malignant Cells.** (Ed.) R. O. Hynes (1979). Chichester: John Wiley & Sons. 471 pp. £24.00

The last decade has seen major advances in our understanding of the physical and biochemical properties of cell membranes. Membranes have gone from 'rigid' to 'fluid', proteins once fixed new float, and the concept that the outside is 'sweeter' than the inside has been reinforced through an overwhelming body of evidence that the carbohydrate portions of surface-membrane glycoproteins are at the outer surface of the cell. This book contains 12 chapters by different authors and describes modern concepts of the biology and biochemistry of the cell surface with particular emphasis on surface changes in malignancy. In his choice of contributors, the editor, Dr Richard Hynes, has struck a nice balance between workers who study deviations in tumour-cell membranes and those whose work illustrates specific aspects of normal membrane structure and function which could be of special relevance to malignancy.

A good overall standard of presentation has been maintained throughout the book and certain chapters are of outstanding quality. I would single out the editor's introductory chapter for the excellent perspective it provides of the tumour-cell problem and for emphasizing the care needed in selecting appropriate control cells. Hynes is not quite so inspired in his main chapter on 'Proteins and Glycoproteins' though most of the relevant literature is critically surveyed and there can be no underestimating the profound impact of his own major contribution to this field with the discovery of LETS protein. An account of the 'Morphology of Normal and Transformed Cells' by Allred and Porter is a gem, taking the reader into the electron microscopic world of blebs, ruffles and various 'podias', all beautifully illustrated, with their morphological complexity showing how little we still know of the molecular basis of this cell-surface wonderland. Surface mucopolysaccharides are comprehensively discussed in an authoritative chapter by Paul Kraemer, who includes some interesting speculation on mucopolysaccharide functions. Glycolipids have undergone a troubled history in the context of surface membranes, 'peaking-out' in popularity in the late sixties/early seventies with the observations of simplification of glycolipid structure in tumour cells, declining in the mid-seventies as important exceptions to the general rule were noted, but now making a comeback as membrane receptors. In his glycolipid chapter, David Critchley gives an interesting account of recent glycolipid history and terminology and discusses enthusiastically his future hopes for these once-famous molecules. Two chapters concern the importance of surface glycoproteins and proteins in the adhesive specificity of embryonic cells and in the development of cellular slime mould; steady progress is being made in these fields but the vital work on 'non-immunological' cellular recognition in adult mammalian tissues still lags behind the embryo and the 'mould'. The editor could not resist a chapter on erythrocyte surfaces, despite extensive documentation elsewhere. Nevertheless, a good account awaits the reader, especially as functional properties of membrane glycoproteins are emphasized. A discussion of functional aspects is also a prominent feature of a chapter by Rozengurt on 'Early Events in Growth Stimulation' with emphasis on the concerted nature of membrane changes that accompany the proliferative response. A chapter by N. Hogg on C-type viruses skilfully blends the terminology of the virologist and immunologist to leave me somewhat catatonic but for those mutual friends who are into MuLV's, VEA's, FMR's, FOCMA's and FeLV's, ecstasy awaits! The book also includes comprehensive reviews on surface-membrane enzymes and on the significance of proteases, particularly plasminogen activator, in tumour cells.

It is clear that many facets of the tumour-cell membrane are covered in this book. I felt the main deficiencies were the failure to include an account of surface markers in leukaemia, as they are particularly useful guides to the stage of maturity or differentiation of tumour cells, and the lack of a separate chapter on lectins, very important historically in emphasising the role of surface membranes in transformation, which would have provided an interesting extra dimension. Nevertheless, this is a valuable book. There are few spelling or grammatical errors, although the terms '*th*umour' and poly*s*eptide' enter the scientific literature.

The book can be recommended to workers in the cell-surface field and to final-year honours students in biochemistry and cell biology, as sufficient background material is provided by most authors. It should provide a stimulus for generating new ideas and also act as a stable point of reference in a rapidly expanding area of research.

J. GALLAGHER